# Anti-nucleolin aptamer AS1411: an advancing therapeutic

**DOI:** 10.3389/fmolb.2023.1217769

**Published:** 2023-09-21

**Authors:** Alexander Van den Avont, Neelam Sharma-Walia

**Affiliations:** Rosalind Franklin University of Medicine and Science, North Chicago, IL, United States

**Keywords:** AS1411, nucleolin, targeted therapy, cancer, endocytosis, pathogens

## Abstract

Targeted therapy is highly desirable, as it allows for selective cytotoxicity on diseased cells without off-target side effects. Nucleolin is a remarkable target for cancer therapy given its high abundance, selective presence on the plasma membrane, and multifaceted influence on the initiation and progression of cancer. Nucleolin is a protein overexpressed on the cell membrane in many tumors and serves as a binding protein for several ligands implicated in angiogenesis and tumorigenesis. Nucleolin is present in the cytoplasm, nucleoplasm, and nucleolus and is used by selected pathogens for cell entry. AS1411 is a guanosine-rich oligonucleotide aptamer that binds nucleolin and is internalized in the tumor cells. AS1411 is well tolerated at therapeutic doses and localizes to tumor cells overexpressing nucleolin. AS1411 has a good safety profile with efficacy in relapsed acute myeloid leukemia and renal cell carcinoma producing mild or moderate side effects. The promising potential of AS1411 is its ability to be conjugated to drugs and nanoparticles. When a drug is bound to AS1411, the drug will localize to tumor cells leading to targeted therapy with fewer systemic side effects than traditional practices. AS1411 can also be bound to nanoparticles capable of detecting nucleolin at concentrations far lower than lab techniques used today for cancer diagnosis. AS1411 has a promising potential to change cancer diagnoses and treatment.

## 1 Introduction

Aptamers are a class of oligonucleotides with unique three-dimensional folding allowing them to be assembled into supramolecular multi-component structures to recognize and bind specific proteins (Keefe et al., 2010; He et al., 2020). As aptamers are generally formed from short single-stranded oligonucleotides (DNA or RNA), they can more easily enter and localize to their targets than immunoglobulins due to their smaller size (10–100 nucleotides) and diminished induction of the immune response (Kumar et al., 2020). Aptamers are produced chemically in a readily scalable process, and their production is not prone to viral or bacterial contamination. Aptamers are small, non-immunogenic, high-binding affinity molecules that can efficiently enter biological compartments. One of the strengths of aptamers is specificity and selection for specific cell-surface targets (Kelly et al., 2021). Some aptamers with guanosine (G)-rich sequences can adopt, in conjunction with metal ions, peculiar secondary structures named G-quadruplexes (G4s) (Tong et al., 2022). The methodology known as Systematic Evolution of Ligands by Exponential enrichment (SELEX), invented around 1990, is a technique used to identify specific oligonucleotides with high affinity and selectivity for their target (Ellington and Szostak, 1990; Tuerk and Gold, 1990). The advances of SELEX technology has led to more efficient and cost-effective development of specific and stable aptamers (Darmostuk et al., 2015; Guo et al., 2008; Catuogno and Esposito, 2017; Kohlberger and Gadermaier, 2022; Chen et al., 2023). The first aptamer for therapeutic use in humans, pegaptanib, was approved in 2004 by the FDA for use in wet age-related macular degeneration (Takeda et al., 2007). There is an excellent potential for use in healthcare treating diseases and research communities of aptamers, which are therefore investigated with numerous goals. The small size of aptamers sometimes makes them susceptible to renal filtration, leading to a shorter half-life. Conjugation chemistries for the attachment of dyes or functional groups can readily be introduced during aptamer synthesis (Healy et al., 2004). Unmodified aptamers are highly susceptible to degradation in serum but conjugated (polyethylene glycol or cholesterol) aptamers rather have increased the circulating half-life and nuclease resistance (Kang et al., 2007). Aptamer technologies have been optimized for activity and persistence under physiological conditions during selection (Guo et al., 2008; Catuogno and Esposito, 2017; Kohlberger and Gadermaier, 2022; Chen et al., 2023). Advancements in aptamer research have allowed sensitive pH responses to enhance control over molecular devices, and improve their diagnostic and therapeutic efficacy (Wilson et al., 2019; Thompson et al., 2020). Aptamers have been, and are currently being, studied in numerous clinical trials. Table 1 shows a selection of results from clinical trials involving aptamers. One of the most promising aptamers is AS1411, formerly AGRO100, now also known as ACT-GRO-777 (Bates et al., 2017). AS1411 is a natural 26-mer (G)-rich DNA [5′-d (GGT GGT GGT GGT TGT GGT GGT GGT GG)-3′]. It is among the aptamers most extensively studied, and is used as an anti-nucleolin drug (Wu et al., 2013).

**TABLE 1:** As per a search on clinicaltrials.gov on 7/1/2023, 35 clinical studies have been done using aptamers. The studies concluded with available data have been included in the table above. Numerous studies are recruiting participants or have been completed and are awaiting published data. These studies carry a wide breadth, including applications in COVID-19 diagnostics, measuring HIV-PrEP compliance, cancer detection, anticoagulation systems, and stem cell transplantation.

Study Title	Patient Population	Intervention	Metric	Outcome	Source
A Study to Establish the Safety and Tolerability of Zimura® (Anti-C5 Aptamer) in Combination with Anti-VEGF Therapy in Subjects with Idiopathic Polypoidal Choroidal Vasculopathy (IPCV) **(Phase 2)**	4 subjects with idiopathic polypoidal choroidal vasculopathy who had prior treatment with anti-VEGF monotherapy	Patients were given monthly intravitreous injections of the anti-C5 aptamer in combination with anti-VEGF therapy	-Number of participants with > 15 Early Treatment of Diabetic Retinopathy Study (ETDRS) letter loss at 3 months -Number of participants with ophthalmic adverse events -Number of participants with systemic adverse events	-0/4 had >15 ETDRS letter loss at 3 months -Average of 2.75 micrometer decrease in retinal thickness -No change in polyps at month 3 -3/4 subjects had conjunctival hemorrhage -1/4 subjects had endophthalmitis	NCT02397954
A Safety and Efficacy Study of E10030 (Anti-PDGF Pegylated Aptamer) Plus Lucentis for Neovascular Age-Related Macular Degeneration **(Phase 2)**	449 patients with subfoveal choroidal neovascularization due to age-related macular degeneration (AMD)	-Lucentis alone -E10030 low dose plus Lucentis -E10030 high dose plus Lucentis	-Change in visual acuity from baseline at 24 weeks -Proportion of subjects that gained 15 ETDRS letters from baseline at week 24 -Proportion of patients with at least 1 adverse event	-Visual acuity in ETDRS letters (Lucentis 6.5; Low dose 8.8; high dose 10.6) -ETDRS letters gained (Lucentis 34; low dose 33.3; high dose 39.1) Adverse events (Lucentis 65.5%; Low dose 67.1%; high dose 65.1)	NCT01089517
A 24 Month Phase 2A Open Label, Randomized Study of Avastin®, Lucentis®, or Eylea® (Anti-VEGF Therapy) Administered in Combination with Fovista® (Anti-PDGF BB Pegylated Aptamer) **(Phase 2)**	60 subjects that have active subfoveal choroidal neovascularization due to age-related macular degeneration	- Fovista® plus bevacizumab intravitreal injection - Fovista® plus ranibizumab intravitreal injection - Fovista® plus aflibercept intravitreal injection	-Total number of systemic adverse events in 2 years -Total number of other adverse Events	-This study was terminated before its completion -Adverse events before termination (Fovista® plus bevacizumab 71.4%; Fovista® plus ranibizumab 76.2%; Fovista® plus aflibercept 76.2)	NCT02387957
Phase 2A Open Label Safety Study of Fovista® (Anti-PDGF BB) Regimen Administered in Combination with Anti-VEGF Therapy to Study Sub-Retinal Fibrosis in Neovascular AMD **(Phase 2)**	101 subjects with all fluorescin angiographic subtypes with presence of active choroidal neovascularization in age-related macular degeneration	- Fovista® (anti-PDGF BB) plus anti-VEGF as a "Pre-Treatment" regimen - Fovista® (anti-PDGF BB) plus anti-VEGF as a "Simultaneous" regimen	-Total number of systemic adverse events in 2 years -Total number of other adverse Events	-Non-serious adverse events (Pretreatment 89.5%; Simultaneous 81%) -Serious adverse events (Pretreatment 21.1%; Simultaneous 30.2%)	NCT02214628
A Phase 2/3 Randomized, Double-Masked, Controlled Trial to Assess the Safety and Efficacy of Intravitreous Administration of Zimura™ (Anti-C5 Aptamer) in Subjects with Geographic Atrophy Secondary to Dry Age-Related Macular Degeneration **(Phase 2 & 3)**	286 Subjects with non-foveal geographical atrophy secondary to dry age-related macular degeneration	-Part 1 (Low Dose, high dose, sham) -Part 2 (High dose + sham, high dose + high dose, sham + sham) -During all parts patients were injected monthly for 18 months	-Change in geographical atrophy (GA) from baseline to months 12 -Change in best corrected visual acuity	-Change in atrophy combined part 1 & 2 (Low dose .292, High dose .321, sham - .402) -Change in visual acuity combined part 1 & 2 (Low dose -7.90, High dose -3.79, Sham -9.29)	NCT02686658
A Phase 3 Safety and Efficacy Study of Fovista® (E10030) Intravitreous Administration in Combination with Lucentis® Compared to Lucentis® Monotherapy **(Phase 3)** **There are 2 similar Stage 3 studies on E10030 that were terminated (NCT01940900 and NCT01940887)	622 subjects with active subfoveal choroidal neovascularization secondary to age-related macular degeneration	-E10030 plus ranibizumab -Sham plus ranibizumab	-Change in visual acuity from baseline to 12 months -Adverse Effects	-This study was terminated before everyone completed the treatment course (The serious adverse events were 15.48% in treatment group compared to 11.65% in the control. The nonserious event rate was 39.68% in treatment vs 36.89% in control) -There was no statistically significant difference in visual acuity between the groups	NCT01944839

As per a search on clinicaltrials.gov on 7/1/2023, 35 clinical studies have been done using aptamers. The studies concluded with available data have been included in the table above. Numerous studies are recruiting participants or have been completed and awaiting published data. These studies carry a wide breadth, including applications in COVID-19 diagnostics, measuring HIV-PrEP compliance, cancer detection, anticoagulation systems, and stem cell transplantation.

### 1.1 AS1411 structure

AS1411 is a 26-base guanine-rich oligodeoxyribonucleotide aptamer that forms a G-quadruplex structure with many beneficial characteristics, notably its ability to bind to nucleolin. AS1411 was not developed by SELEX but discovered by chance (Bates et al., 2009). AS1411 targets cells with higher concentrations of nucleolin on the cell membrane and in the cytoplasm–a characteristic of many cancer cells and pathogen-infected cells. When in the proper pH, with available cationic metals to stabilize the structure, the guanines form Hoogsteen bonds forming G-tetrads; stacking two or more G-tetrads gives G-quadruplex structures that can assume several conformations. Depending on various factors, including the metal ion available, the DNA strands configure into parallel, anti-parallel, or mixed structures (Tong et al., 2022). The names of the G4 structures come from the orientation of the strands. Potassium provides the most stable and common G-quadruplex structures in the parallel conformation (Kosiol et al., 2021). There are 26 possible looping topologies and eight possible tetrad arrangements of AS1411; this means that a single AS1411 G4 complex can theoretically have 208 unique unimolecular conformations (Dailey et al., 2010).

At temperatures near or exceeding the Tm of 67°C, the aptamer can change its conformation to reach an equilibrium among different species. At lower temperatures, the concentration of the unfolded AS1411 is infinitesimal, thus leading to an equilibrium that may differ from the expected (Dailey et al., 2010). These properties create the need to quantify the structural makeup of an AS1411-containing solution. In 2010, Lane and Trent’s group deeply investigated AS1411 and discovered its high polymorphism in complex mixtures of different G4 structures. The behavior of AS1411 is not unique, and techniques such as circular dichroism, electrophoresis, UV melting, CD melting, and analytical ultracentrifugation are insufficient in fully characterizing the different conformations of AS1411 fully. If the exact structure of AS1411 is needed, high-resolution techniques like NMR are essential (Dailey et al., 2010). The G4 structures are very stable to heat and serum nucleases; they also preferentially enter cancer cells without permeating the cell membrane or causing damage to normal cells (Ogloblina et al., 2020; Saravanakumar et al., 2020).

AS1411 is the first anticancer “G-4 forming GRO” with good biological activity, reaching phase II clinical trials for acute myeloid leukemia and renal cell carcinoma (Bates et al., 2009; Rosenberg et al., 2014). Antiproliferative biological effects of AS1411 are due to its interaction with nucleolin and various nucleolin-independent effects (Bates et al., 2017; Ogloblina et al., 2018). The highlight of the clinical development of AS1411 was not only its anticancer and antiproliferative effects, but its ability to be loaded with several drugs that allow for the delivery of that drug to a specific target cell. AS1411 can also be linked with biomarkers that allow identification of cells with a high level of nucleolin (Bie et al., 2022; Tong et al., 2022). These advancements will be discussed in much detail throughout this review.

In 2021, the Miranda group developed a derivative of AS1411, termed AS1411-N6 (Miranda et al., 2021). AS1411-N6 had 6 nucleotides added at the 5′ end of the sequence of AS1411, forcing the aptamer to fold into a stem-loop structure. This new conformation decreases the structural polymorphism of the G4-forming aptamer; when AS1411-N6 aptamer is in the presence of potassium ions, it forms a unique duplex/G4 hybrid structure. The appeal of AS1411-N6 is that when loaded with a fluorescence marker, a 3-fold increase in the fluorescence at saturation nucleolin concentrations is detected (Miranda et al., 2021).

Other AS1411 derivatives include analogues 5′-conjugated with lipophilic tails (Riccardi et al., 2018). These lipid-conjugated aptamers have applications in liposomal formulations and lipid coated-nanoparticles for targeted cancer therapies, and these aptamers fold into stable, parallel unimolecular G-quadruplex structures, forming large aggregates like micelles (Riccardi et al., 2018). AS1411 has also been used as a supramolecular carrier for the cancer-selective delivery of acridine orange derivatives (C_3_, C_5_, and C_8_), especially in cervical cancer (Figueiredo et al., 2019). This delivery strategy relied on the non-covalent association of the acridine derivatives and the G4 structures (Figueiredo et al., 2019).

### 1.2 Nucleolin

Nucleolin is a ubiquitous multifunctional protein discovered in 1973 and was initially called C23. As its name suggests, nucleolin is a protein predominately found in the nucleolus, nucleoplasm, cytoplasm, and cell membrane (Orrick et al., 1973; Jing et al., 2020). Nucleolin consists of three main structural domains: the N-terminal domain, the central domain, and the C-terminal domain. The N-terminal domain is rich in acidic amino acids and is the predominant site of phosphorylation during the cell cycle (Peter et al., 1990; Ginisty et al., 1999); it participates in the transcription of rRNA and interacts with the components of the pre-rRNA processing complex. Extensive phosphorylation by casein kinase 2 (CK2) occurs at interphase and by cell division control protein 2 homolog **(**CDC2) during mitosis; the regulated phosphorylation of nucleolin regulates its role during the cell cycle (Khurts et al., 2004). The central domain contains four RNA-binding domains (RBD) (also called RNA-recognition domains) that predominately aid in producing rRNA by acting as a chaperone and a pre-ribosome component. The central domain also aids in the biological packaging of RNA, pre-mRNA splicing, and poly-A tail synthesis. The RBDs also interact with telomerase and are believed to be the localization mechanism to the nucleolus (Khurts et al., 2004). The C-terminal domain is rich in Arg-Gly-Gly repeats that lead to the unique structure of the C-terminus; this leads to interactions with mRNA and facilitates the interactions of RBDs and large RNA. The C-terminus is also essential for assembling the ribosome and importing ribosomal proteins into the nucleus (Jia et al., 2017). Although nucleolin lacks a transmembrane domain or signal sequence, nucleolin is nevertheless present on the surface of various cell types (Chen et al., 2008).

To function correctly, nucleolin has to travel between the nucleus, cytoplasm, and plasma membrane. Nucleolin can be modified to facilitate its function and shuttling; it can be phosphorylated, methylated, and ADP-ribosylated (Kang et al., 1975; Lischwe et al., 1982; Leitinger and Wesierska-Gadek, 1993). While nucleolin’s function is well-documented and summarized above, its RBD domain is found to be reductive. The knockout of nucleolin’s central functional unit, RBD, does not affect the transcription, rRNA maturation, and shape in chicken B-lymphocytes (Storck et al., 2009). They did find that nucleolin is required for the proliferation and survival of cancer cells (Storck et al., 2009). These data are an excellent example of why nucleolin is a promising target for cancer treatment, its inhibition in cancer cells limits the ability to grow and spread while not impacting the normal function of non-cancer cells.

Nucleolin is overexpressed in non-small cell lung, gastric, ovarian, breast, and kidney cancers (Masiuk et al., 2007; Cicchillitti et al., 2009; Qiu et al., 2013; Rosenberg et al., 2014; Xu et al., 2016). It can be found on the cell surface of HeLa cells, lymphoblastoid T-cells, breast carcinoma, hepatocarcinoma, laryngeal epithelial cells, and endothelial cells of angiogenic blood vessels (Fogal et al., 2009; Fujiki et al., 2014; Koutsioumpa and Papadimitriou, 2014; Niu et al., 2015). Viruses such as Human immunodeficiency virus-1 (HIV-1), Respiratory syncytial virus (RSV), Influenza A, Parainfluenza virus 3, and Enterovirus 71 have all been shown to use nucleolin to enter a human host cell (Bose et al., 2004; Su et al., 2015; Chan et al., 2016; Perrone et al., 2016; Mastrangelo et al., 2021). HSV-1, Rabies virus, Epstein-Barr virus (EBV), hepatitis C virus (HCV), Dengue virus, and bacterial infection *F.tularensis* have shown to use nucleolin in part of their life cycles (Shimakami et al., 2006; Barel et al., 2008; Callé et al., 2008; Balinsky et al., 2013; Oksayan et al., 2015; Lista et al., 2017). Numerous studies cited above have shown that inhibition of nucleolin leads to decreased vitality of corresponding cancers and viral infections. Nucleolin has a promising potential as a therapeutic target that can be used in diagnosis and treatment.

There is the promise that AS1411 might have potential in other viruses based on work done on other G4 structures. The Verma lab showed that G4 structures inhibit Kaposi’s sarcoma herpesvirus latency-associated nuclear antigen (KSHV-LANA) mRNA translation, a major latency-associated gene (Dabral et al., 2020). Our lab conducts an IACUC-approved *in vivo* study on AS1411 to treat KSHV+ Primary Effusion Lymphoma (PEL) in NOD/SCID mice. The mice are injected with 5 × 10^6^ KSHV + PEL cell line BCBL-1 cells to induce tumorigenesis. They are then either injected with AS1411 or the control [5′-d (CCT CCT CCT CCT TCT CCT CCT CCT CC)-3′] and monitored for survival and tumor growth; after the mice have humanely euthanized, their tumors, spleens, and ascites are collected for further lab evaluations. This is one example of how AS1411 can be used experimentally in oncogenic viral infected-cell tumors. Numerous other *in vivo* studies and their findings are highlighted in Table 2.

**TABLE 2:** A selection of publications citing non-clinical experiments using AS1411 conjugated to (a) nanoparticle(s). Each of these studies has an application in treatment, or detection of a disease state in humans..

Conjugate	Target Disease	Diagnostic/ Therapeutic	Notes	Source
Doxorubicin-Bovine serum albumin	Breast Adenocarcinoma	Treatment	AS1411 was attached to Doxorubicin, this conjugate was then loaded onto Bovine serum albumin. This complex localized to cells overexpressing nucleolin leading to the downregulation of anti-apoptotic protein B-cell leukemia/lymphoma 2 protein (Bcl-2) and proliferating cell nuclear antigen (PCNA). It led to the upregulation of proto-oncogenic tumor suppressor retinoblastoma protein (pRB), DNA repair protein poly ADP-ribose polymerase (PARP), and an inducer of apoptotic cell death Bcl-2-associated X protein (Bax)	Xu et al. (2018)
miR-145 - Dextran	Breast Adenocarcinoma	Treatment	AS1411 was decorated on the surface of miR-145 (tumor suppressive) conjugated thiolated dextran. This complex specifically targeted nucleolin-expressing MCF7 Breast Adenocarcinoma cells and delivered the cargo to the cytoplasm.	Tekie et al. (2018)
C8 ligand	Malignant Melanoma	Treatment	In a murine model, AS1411 conjugated with C8 ligand increased cytotoxicity in the murine melanoma B16 cell line. C8 increased the thermostability of AS1411 without affecting its G4 topology.	Lopes-Nunes et al. (2020)
Doxorubicin	Breast Adenocarcinoma	Treatment	Doxorubicin was loaded onto α-PCNA and then condensed and coated with AS1411. This complex accumulated and is internalized at the tumor site. The targeted cells showed enhanced anticancer activity compared to doxorubicin alone.	Taghdisi et al. (2020)
Polyethylene glycol-manganese oxide	Renal Carcinoma	Diagnostic	AS1411 was conjugated with polyethylene glycol-MnO for imaging with T1 weighted MRI. This complex was able to clearly visualize renal carcinoma cells in vitro.	Li et al. (2018)
Streptavidin	Wilson’s/Alzheimer’s/Diabetes Mellitus	Diagnostic	AS1411-Streptavidin conjugate was able to detect Cu++ levels of .01 µM in serum samples of healthy patients compared to those with Wilson’s, Alzheimer’s, and Diabetes Mellitus. AS1411-Streptavidin conjugate, when applied topically to the cornea, could be delivered to the nucleus and cytoplasm of corneal endothelial cells.	Bahreyni et al. (2019)
Doxorubicin-poly D,L-lactic-co-glycolic acid-poly N-vinylpyrrolidone	Lung Adenocarcinoma	Treatment	This complex was able to trigger cell death by activating apoptosis related proteins without harming normal cells in a mouse model. The mice that received the treatment significantly recovered from lung cancer compared to control.	Saravanakumar et al. (2019)
360A	In vitro drug at this time	Treatment	360A (a telomerase inhibitor) was found to be the most promising affinity ligand for both AS1411 and AS1411-N6	Miranda et al. (2021)
Gemcitabine	Non-small cell lung cancer (NSCLC)	Treatment	Gemcitabine loaded AS1411 was decorated with polyethylene glycol-poly (lactic-co-glycolic acid) nanopolymersome. This complex targeted nucleolin-overexpressing NSCLC cells and exhibited a controlled release pattern and internalization of gemcitabine.	Alibolandi et al. (2016)
Doxorubicin	Breast Adenocarcinoma	Treatment	A multifunctional composite micelle was surface loaded with AS1411 modified Pluronic F127 and beta-cyclodextrin-linked poly (ethylene glycol)-b-polyacitide. This complex increased micelle stability, doxorubicin loading ability, micelle uptake, circulation time in blood, accumulation in tumor, antitumor activity, and decreased cardiotoxicity.	Li et al. (2015)
TGN peptide modified nanoparticles (AsTNPs)	Gliomas	Treatment	A system was developed consisting of AS1411 and AsTNPs that allowed for penetration of the blood-brain-barrier and glioma targeting in microenvironmental assays.	Gao et al. (2014)
Doxorubicin-MUC1 aptamer	Breast Adenocarcinoma	Diagnostic/ Treatment	AS1411 was loaded with Doxorubicin as well as an anti-mucin short variant S1 (MUC1) protein aptamer with a fluorophore domain for real-time imaging. This complex showed lower cytotoxicity to normal liver cells than doxorubicin alone.	Liu et al. (2018)
Paclitaxel	Gliobastoma	Treatment	A nanoconjugate system comprising of AS1411-poly (l-γ-glutamyl-glutamine)-paclitaxel was developed that optimizes the solubilization of paclitaxel. In vivo models show that the cellular uptake of paclitaxel was increased, and the median survival time increased in this system compared to paclitaxel in isolation.	Luo et al. (2017)
Doxorubicin-Au nanocluster-cyclic RGD-near infrared fluorescent dye MPA	αvβ3 integrins on tumor cells	Diagnostic/ Treatment	This cyclic RGD conjugate (which binds to αvβ3 integrins), when attached to AS1411 localizes to tumor cells as both αvβ3 integrins and nucleolin are over expressed on tumor cells. The complex penetrates deep tumors and delivers doxorubicin resulting in improved anti-tumor activity in a mouse model. The infrared MPA, as well as the Au nanocluster allows for tumor imaging both *in vitro* and *in vivo*.	Chen et al. (2016)
6-mercapto-1-hexanol	Nucleolin- expressing tumor cells	Diagnostic	AS1411 was fashioned into a microcantilever and then modified with 6-mercapto-1-hexanol. When AS1411 binds nucleolin there is induced surface stress leading to a deflection that can be detected by a sensor. This system allows detection of nucleolin at a limit concentration of 1.0 nM.	Li et al. (2016)
5-Flurouracil	Basal Cell Carcinoma	Treatment	A gel formulation containing AS1411 aptamer-functionalized polymeric nanocapsules loaded with 5-Flurouracil was developed for topical use. The complex was determined to have favorable biosafety as well as increased antitumor effects compared to 5-flurouracil alone.	Rata et al. (2021)
Erlotinib	Non-Small-Cell Lung Carcinoma	Treatment	Chitosan nanoparticles were loaded with erlotinib and then decorated with AS1411 to target non-small-cell lung cancer cells in a pH dependent manor. This complex improved uptake of erlotinib and induced apoptosis through release of reactive oxidative species.	Saravanakumar et al. (2020)
Docetaxel-TGN	Gliomas	Treatment	AS1411 was conjugated with the TGN peptide to form a drug delivery system that can penetrate the endothelial monolayer and the core of tumor spheroids of gliomas. When this system is loaded with an antineoplastic agent Docetaxel, there was high distribution of tumor distribution without much off site delivery as measured by the tumor/normal brain ratio.	Tekie et al. (2018)
Dextran-Graphene Oxide-Curcumin	Mammary Carcinoma/ Breast Adenocarcinoma	Treatment	When AS1411 was conjugated on Graphene Oxide, Dextran, and Curcumin, the complex can effectively enter breast cancer tissue and exert higher cytotoxicity than Graphene Oxide-Dextran alone.	Tekie et al. (2018)
Au@luminol nanoparticles	Circulating Tumor Cells	Diagnostic	AS1411 was added to magnetic nanoparticles in a manner that allowed a hybridization chain reaction to take place. This system results in a response signal that can be sensed at concentrations as low as 3 cells/mL in whole blood samples.	Cao et al. (2020)
.9% NaCl for Solubilization	Wet Age-Related Macular Degeneration	Treatment	AS1411 was solubilized in normal saline for both intravitreal injection, and topical application in mice with wet age-related macular degeneration. Both treatment groups of mice saw a significant decrease in the amount of choroidal neovascularization.	Leaderer et al. (2015)
Rox	Nucleolin-expressing tumors in situ	Diagnostic	Rox, a fluorescent dye, is attached to AS1411 and allowed to form a G4 complex by passing through plasmonic metamaterial decorated with cDNA in a plasmon enhanced Raman scattering detection device (PERS). In the presence of nucleolin in situ, the AS1411-Rox will dissociate and the change in signal can be sensed at a limit as low as 71pM in under 10 min.	Shi et al. (2021)

## 2 Binding and uptake of AS1411

### 2.1 Binding of AS1411 to nucleolin

AS1411 can bind to nucleolin and subsequently enter the cytosol, leading AS1411 to deliver drugs and dyes to cancer cells. Whether inside the cell or on the plasma membrane, AS1411 finds and binds to nucleolin. The central region of nucleolin consists of 4 RBDs. The exact binding mechanism is not fully understood, but Bie et al. (2022) proposed a model in 2022. They proposed that AS1411 undergoes capping at the 5′ end, while the folding of AS1411 into a G4 structure leads to the Thymidine residues facing a groove of nucleolin. The 5′ cap forms the bond with the RBD1 of nucleolin; this binding is stabilized by AS1411 interacting with RBD2 via hydrogen bonds, salt bridges, and a water-mediated network. The binding of AS1411 downregulates nucleolin phosphorylation by blocking the phosphorylation sites near the N-terminus (Iturriaga-Goyon et al., 2021). Loss of the N-terminal phosphorylation leads to the inhibition of transcription of rRNA and interactions with the components of the pre-rRNA processing complex (Jia et al., 2017).

### 2.2 Role of nucleolin in endocytosis

Endocytosis is the broad term for the entry of a substrate into a cell enclosed by its cell membrane resulting in a vesicle. Endocytosis evolutionarily allows cells to accumulate nutrients and resources necessary for survival and remove foreign material from the aqueous environment. Pathogens can hijack this process for their replication and transmission; it can also be used therapeutically for drug delivery and signal transduction (Doherty and McMahon, 2009).

There are four mechanisms by which a cell achieves endocytosis: Phagocytosis, Caveolae, Pinocytosis, and Receptor-mediated endocytosis (also known as clathrin-mediated endocytosis). Phagocytosis is the process of internalizing particles larger than 0.5 μm in diameter; it is a crucial function of many cells in the immune system. Caveolae are non-clathrin-coated invaginations that consist of cholesterol, caveolin, and glycoproteins; they are prominent in smooth muscle, fibroblasts, adipocytes, type I pneumocytes, and endothelial cells (Doherty and McMahon, 2009). In 2010, Bates and Luo’s groups showed that caveolae are not involved in AS1411 nor nucleolin-associated viral uptake in prostate cancer cells, endothelial cells, or skin fibroblasts (Reyes-Reyes et al., 2010; Song et al., 2012).

**FIGURE 1:**
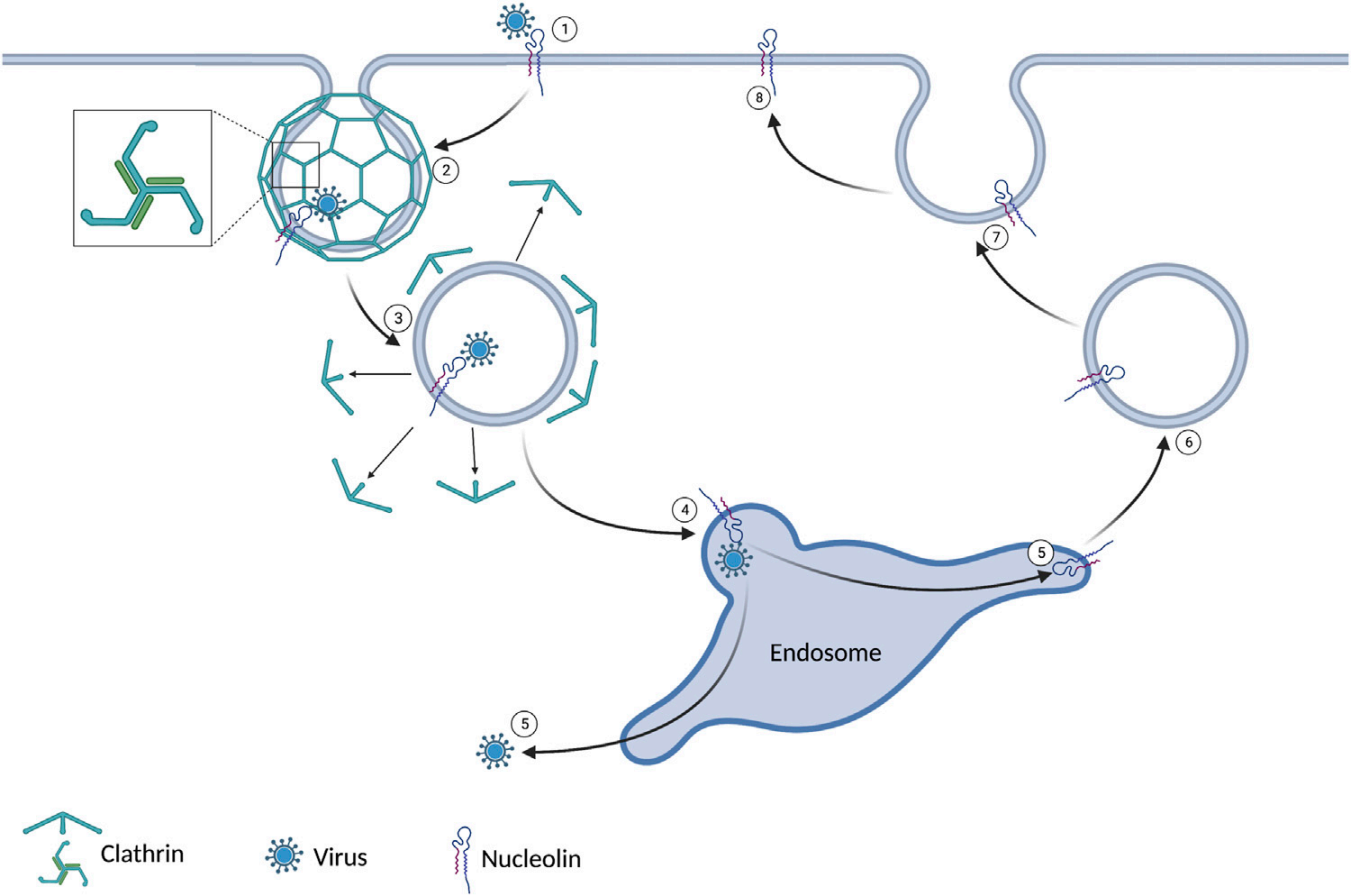
1) The virus binds to nucleolin expressed on the cell surface. 2) Clathrin coats the intracellular plasma membrane around the virus-bound receptor. This leads to an invagination and ultimately a vesicle. 3) After the vesicle gets internalized, the clathrin dissociates. 4) The vesicle joins the endosome. 5) The virus evades the endosome and enters the cytoplasm. 6/7/8) The nucleolin is recycled to the extracellular domain.

Receptor-mediated endocytosis is the primary route for endocytosis in most cells. The cell can take up a known substrate bound to a specific extracellular domain. When the extracellular domain of a clathrin-associated receptor is activated, a conformational change of the cytoplasmic domain is initiated. The conformational change results in clathrin aggregating in the cytoplasm along the plasma membrane. Clathrin has a tri-skeleton structure allowing self-interaction and forming a clathrin cage. The clathrin cage results in a vesicle that contains the receptor, its bound substrate, and a small amount of extracellular fluid. This vesicle will be trafficked to an endosome to deliver its contents before recycling the receptor back to the plasma membrane (Doherty and McMahon, 2009). This process is summarized in Figure 1. One such receptor that utilizes clathrin-mediated endocytosis is nucleolin. Several viruses have utilized this receptor as a means of entry. HIV-1, RSV, Influenza A, Parainfluenza virus 3, and Enterovirus 71 have all been shown to use nucleolin to enter human host cell (Bose et al., 2004; Su et al., 2015; Chan et al., 2016; Perrone et al., 2016; Mastrangelo et al., 2021). Several viruses in non-human hosts also use this mechanism. Studies have also shown that decreased nucleolin levels achieved with siRNA and AS1411 have decreased viral titers in HIV-1, RSV, Influenza A, Parainfluenza virus 3, and Enterovirus 71 (Bose et al., 2004; Su et al., 2015; Chan et al., 2016; Perrone et al., 2016; Mastrangelo et al., 2021).

As nucleolin can act as a receptor for receptor-mediated endocytosis, it can also act as a receptor for phagocytes. Phagocytosis is the process of internalizing particles larger than 0.5 mm in diameter. The first phase of phagocytosis is recognizing the particle to be injected (Uribe-Querol and Rosales, 2020). This can be done with various receptors, one of which is nucleolin. Nucleolin is used in microglia recognition of amyloid beta peptide 1–42 (Ozawa et al., 2013), macrophage recognition of maleyated bovine serum albumin, and acetylated low-density lipoprotein (Miki et al., 2015; Miki et al., 2015), to name a few. After recognition, there is the process of internalization through cytoskeleton rearrangement and, ultimately, degradation (Uribe-Querol and Rosales, 2020).

### 2.3 Proposed mechanism of endocytosis and localization of AS1411

Pinocytosis, coming from the Greek pino-meaning “to drink,” is the process by which a cell forms a vesicle containing solutes in the extracellular fluid. Pinocytosis can be divided into non-specific adsorptive pinocytosis and macropinocytosis. Non-specific adsorptive pinocytosis is a clathrin-mediated process. It differs from receptor-mediated endocytosis in that the clathrin dissociates from the vesicle immediately upon forming. The vesicles commonly fuse with early endosomes to digest their contents (Doherty and McMahon, 2009). Macropinocytosis is a clathrin-independent process that utilizes actin to induce “membrane ruffling,” resulting in the formation of vesicles. This actin remodeling is under tight control via several pathways, including the Ras-related C3 botulinum toxin substrate 1 (Rac1) pathway (Koronakis et al., 2011). Rac1 is a member of the Rho family of GTPases. Positive regulation of Rac1 is obtained through guanine exchange factors (GEF); when a GEF activates Rac1, the result is Rac1GTP. Endogenous activators of Rac1 include serine/threonine p21-activating kinase 1 (PAK1), Ras, phosphoinositide 3-kinase (PI3K), and SRC Proto-oncogene non-receptor tyrosine kinase (Src). Negative regulation of Rac1 results in Rac1GDP, the inactive form of Rac1; nucleolin is a negative regulator of Rac1 (Reyes-Reyes et al., 2015).

**FIGURE 2:**
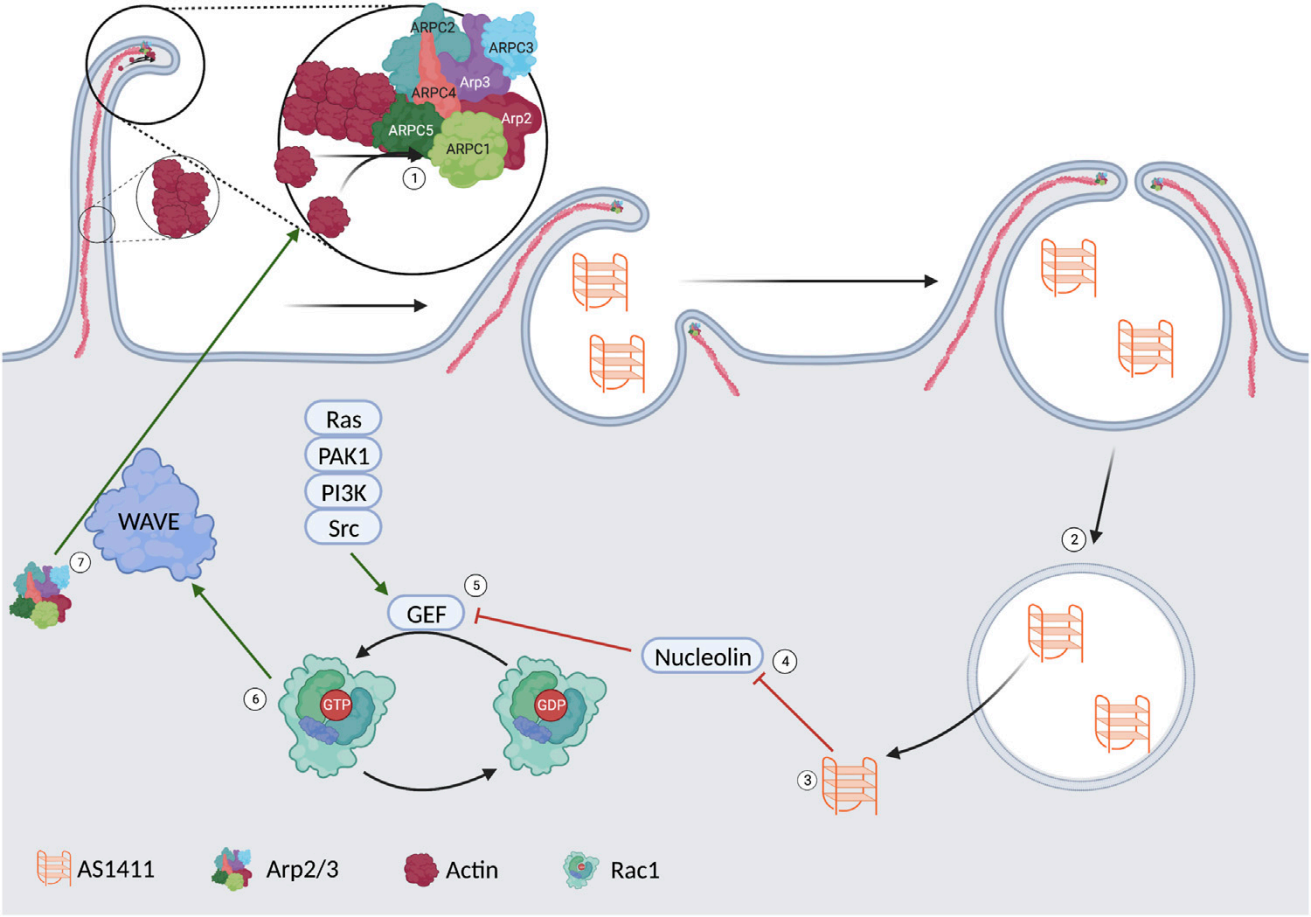
1) Arp2/3 induces actin polymerization leading to membrane ruffling and the formation of a vesicle. 2/3) The AS1411 is released from the vesicle into the cytoplasm. 4) AS1411 leads to the inhibition of nucleolin. 5) The inhibited nucleolin leads to an upregulation of a guanosine exchange factor. 6) The activated GEF leads to the conversion of Rac1GDP to Rac1GTP. 7) rac1GTP leads to activation of the WAVE complex. 7) The WAVE complex upregulates Arp2/3 and more membrane ruffling.

Rac1GTP activates the WASP family verprolin homologous protein (WAVE) regulatory complex (WRC). The WRC is active when phosphorylated, activating Arp2/3, leading to actin polymerization, membrane ruffling, and, thus, endocytosis (Koronakis et al., 2011; Reyes-Reyes et al., 2015). The importance of highlighting this specific pathway is to show nucleolin’s role in cellular uptake. This is represented visually in Figure 2. It is hypothesized that one of the ways AS1411 gets localized to cancer cells lies in the increased level of macropinocytosis found in many cancer lines. This initial increase in extracellular fluid uptake leads to a basilary level of AS1411 entering the cytoplasm. The AS1411 then inhibits nucleolin, leading to an increase in Rac1GTP and, thus, further macropinocytosis in a feed-forward loop in which more AS1411 is taken up by the cell (Reyes-Reyes et al., 2015).

## 3 AS1411 as an anti-nucleolin aptamer

AS1411 began being studied because of its ability to bind nucleolin. It was soon discovered that AS1411 significantly decreased the survivability of cancer cells expressing nucleolin. The specific role that AS1411 (and by effect nucleolin) plays in these pathways is yet to be clarified for many cancer-cell lines. For example, the Cheng et al. (2016) explored the expression level of several factors in glioma cells. They found that when glioma cells were treated with AS1411, there was an upregulation of tumor suppressor gene p53 and a downregulation of oncogenes Bcl-2 and Akt1. These findings were discovered when nucleolin was silenced, suggesting that these results were the effect of AS1411 directly inhibiting nucleolin. These authors cited the 2010 work of the Ishimaru group to propose a complete mechanism of action. Without AS1411, nucleolin’s RBD will bind to the 5′ UTR of B-cell Leukemia/lymphoma Protein 1 (Bcl-1) mRNA and the 5′ UTR of p53 mRNA. Binding to Bcl-1 promotes expression, while binding to p53 decreases expression. The decreased concentration of Akt1 is attributed to it being downstream of a Bcl-1 pathway (Ishimaru et al., 2010). AS1411, 5′ UTR of Bcl1 mRNA, and 5′ UTR of p53 mRNA all bind to the exact location; it is hypothesized that AS1411 acts as a competitive inhibitor for these targets.

The above mechanism was the basis for the hypothesis of using AS1411 alone as an anti-cancer drug. Using AS1411 alone to treat solid tumors has been investigated. In 2007, a phase I clinical trial was completed on 30 adult patients with advanced solid tumors to determine the maximum tolerated dose. In 2010, a phase II clinical trial was started, studying 35 patients with metastatic, clear-cell, renal cell carcinoma after only 2.9% reported a response (Rosenberg et al., 2014). No other studies in humans use AS1411 alone as of May 2023.

**FIGURE 3:**
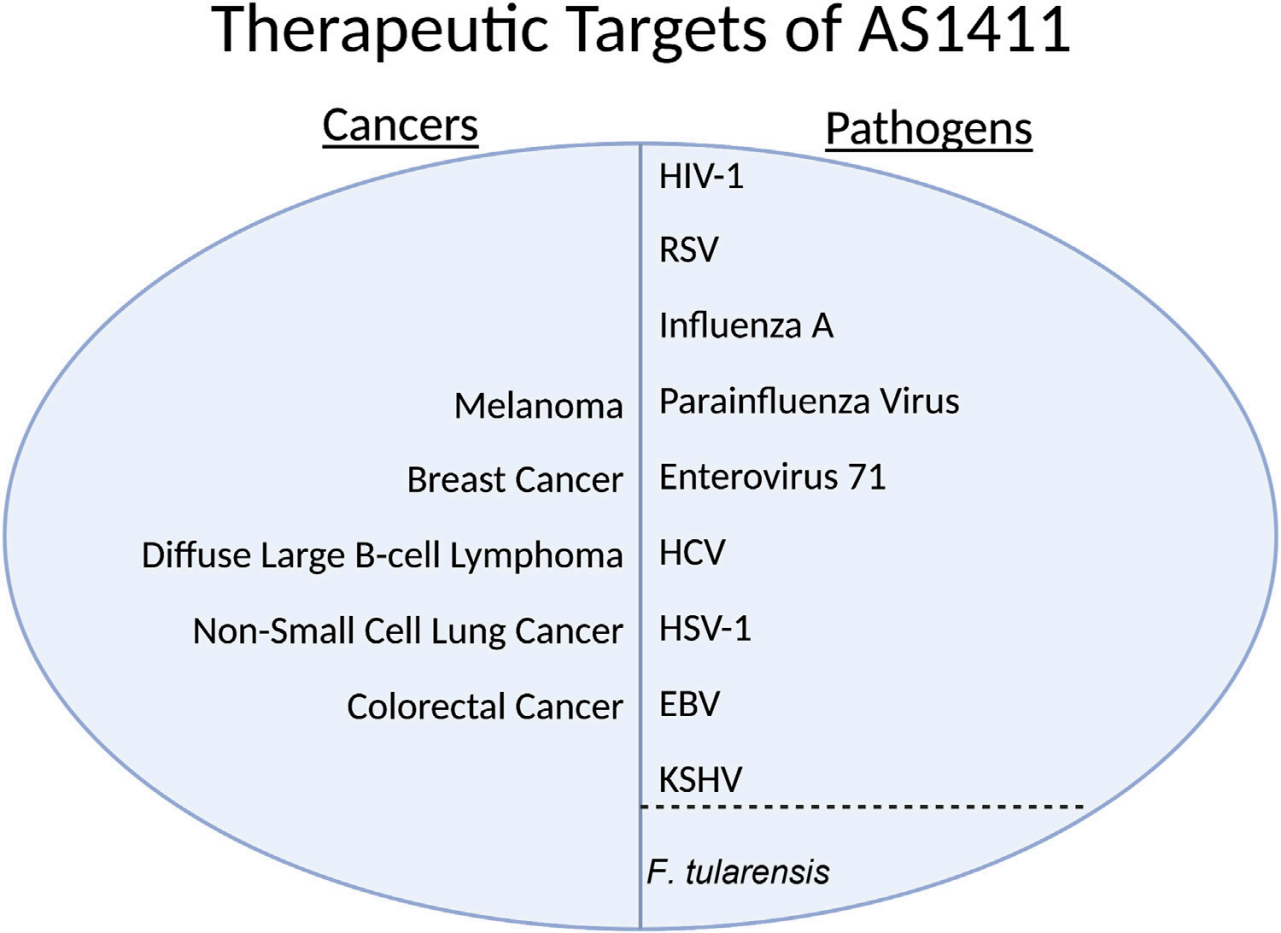
HIV-1, Human Immunodeficiency Virus 1; RSV, Respiratory Syncytial Virus; HCV, Hepatitis C Virus; HSV-1, Herpes Simplex Virus 1; EBV, Epstein-Barr Virus; KSHV, Kaposi’s sarcoma-associated herpesvirus.

In addition to nucleolin’s role in ribosome maturation and interacting with the cell cycle intermediaries CK2 and CDC2, nucleolin also exists on the extracellular side of the plasma membrane. HIV-1 has a low-level affinity to the extracellular domain of nucleolin. Perrone et al., 2016 demonstrated that treatment with AS1411 can limit the entry of HIV-1 into the host cell *in vitro* (Figure 3) (Perrone et al., 2016). Respiratory Syncytial Virus (RSV) has been shown to use nucleolin as a functional receptor that passively interacts with fusion proteins (F proteins) (Figure 3). F proteins are glycoproteins on the virion surface and are crucial in the initial phase of infection (McLellan, Ray, and Peeples, 2013). Mastrangelo et al. (2021), in 2021 demonstrated that AS1411 could inhibit endocytosis and decrease the number of RSV (+) cells *in vitro* and mouse and rat models at a non-toxic level. The hemagglutinin glycoprotein found on the surface of Influenza A has been shown to bind to nucleolin in addition to sialic acid (Figure 3).

Chan et al. (2016), in 2016 showed that inhibiting nucleolin with a specific nucleolin antibody, blocking the virus with purified protein, and siRNA knockdown all substantially decreased the internalization of several virus strains in lung endothelial cells *in vitro* (Figure 3). Bose, Basu, and Banerjee (2004) in 2004 showed that Parainfluenza virus type 3 requires nucleolin expression on the cell surface for efficient cellular entry into lung epithelial cells *in vitro*; they accomplished the knockdown via treatment with an anti-nucleolin antibody and preincubation with nucleolin (Figure 3). Enterovirus-71’s (EV71) capsid protein VP1 interacts directly with nucleolin expressed on the cell surface. Su et al., 2015 showed that cells treated with an anti-nucleolin antibody reduced the binding of EV71 to human cells while knockdown decreased binding, infection, and virus production (Figure 3). They also conferred the nucleolin gene in the mouse model; mice with human-nucleolin expressed on their cells conferred EV71 infection and production (Figure 3) (Su et al., 2015). In HSV-1, nucleolin is required for an efficient infection through its suspected involvement in the HSV-1 DNA replication process via ICP4 (Figure 3) (Callé et al., 2008).

In the Rabies virus, nucleolin interacts with isoform P3 (Figure 3). Depletion of nucleolin leads to decreased protein expression and lower viral production in infected cells (Oksayan et al., 2015). In the Dengue virus, nucleolin plays a role in viral morphogenesis by interacting with viral capsid protein before spread (Figure 3) (Balinsky et al., 2013). In HCV, nucleolin is required to interact with NS5B for RNA-dependent RNA polymerase to function (Figure 3) (Shimakami et al., 2006). The Lista group in 2017 showed that in EBV, nucleolin expression levels are unique, as the virus uses nucleolin to evade the immune response (Figure 3). The viral protein Epstein-Barr virus nuclear antigen 1 (EBNA1) is a soluble protein crucial for the virus to establish and maintain infection; it is only functional in its free form. It is highly antigenic to the human immune system. The viral mRNA for EBNA1 binds to nucleolin, limiting the immune response to the viral mRNA; however, the mRNA bound to nucleolin cannot be translated.

Thus, the virus must exist in an environment with a nucleolin concentration rather similar to the standard concentration of a normal cell. Too little nucleolin and the immune system will mount a response; too much nucleolin and there will be an insufficient amount of EBNA1 protein. This use of nucleolin has yet to be seen in other viruses that infect humans (Lista et al., 2017). In the bacteria *F.tularensis*, it has been shown that nucleolin binds to bacterial ligand elongation factor-thermo-unstable (EF-Tu), aiding its adhesion and entry process. EF-Tu, a vital protein in *F.tularensis* translates bacterial proteins (Figure 3) (Barel et al., 2008).

## 4 AS1411s ability to bind modified particles

The functional aspect of the G4-forming AS1411 lies in the central region. This allows the 5′ and 3′ tails of AS1411 to be modified without resulting in a decreased affinity for nucleolin. One such modification is a thiol modification that allows AS1411 to be linked to drugs/biomarkers via a covalent sulfide bond at either the 5′ or 3′ end (Pon and Yu, 2004). Riccardi et al. (2018) studied three derivatives of AS1411 with different lipophilic tails attached to the 5′ domain of the aptamer. Their studies in pseudo-physiological buffers that mimic extra- and intracellular environments showed that the ability of AS1411 to maintain its G4 motif is unaffected by lipophilic tails. This study showed that these lipophilic tails decreased the cytotoxicity of AS1411, so it is unlikely to be used in cancer therapeutics; however, it demonstrates the ability of AS1411 to maintain its G4 complex (and thus its nucleolin binding ability) while conjugated. Like lipid tails, AS1411 has also been conjugated to niosomes for targeted delivery to cancer cells. Niosomes are vesicles made of non-ionic surfactants that can be conjugated with lipids to deliver hydrophilic and lipophilic drugs (Riccardi et al., 2018). The size of the groups attached to AS1411 can vary significantly and, in many cases, dwarf AS1411 without affecting its ability to bind nucleolin. The consequence of this capability is that the nanoparticle attached to AS1411 is preferentially delivered to where nucleolin is, with limited off-target binding, a valuable feature in cancer/virus therapeutics and diagnosis (Wandtke, Woźniak, and Kopiński, 2015; Tong et al., 2022).

Some of the major complications of traditional chemotherapeutics are the systemic effects of off-target delivery. Significant advancements have been made utilizing the increased extracellular expression of nucleolin in cancer cells. When AS1411 is loaded with an anti-cancer drug, the medication will be preferentially found in upregulated nucleolin-expressing cells. Most particles conjugated to AS1411, per a thorough PubMed review, consist of established drugs/markers often linked in series. An example is the delivery of orange derivative (C_8_) and Imiquimod to gynecological carcinoma cells from the Lopez-Nunes group. They could use gold nanoparticles to covalently conjugate these particles to AS1411, intracellularly allowing on-target dissociation. This conjugate was delivered using a topical gel comprised of polyethylene glycol that could be applied directly to the female genital tract. Their study showed decreased viability of the cancer cells in their porcine model (Lopes-Nunes et al., 2020). The Carvalho group demonstrated that AS1411 can be valuable therapeutically when bound to lipids. These AS1411-lipid conjugates form nano aggregates suitable for pharmaceutical applications due to their small size, negative charge, and drug release capabilities. They demonstrated these findings by seeing a decrease in HeLa cell proliferation and increased cell uptake (Carvalho et al., 2021). The Riccardi et al., 2018 group showed that when AS1411 is conjugated to a niosome it can deliver ruthenium (III) complexes to HeLa cells. This model demonstrated increased antiproliferative activity when the niosomes were decorated with AS1411 than with the control.

A similar idea can be used if AS1411 is loaded with a biomarker and exposed to a sample suspected of cancer; a greater level of AS1411 bound can be highly suggestive of a positive result (Tong et al., 2022). In viral infections, similar patterns might be expected for treatment and diagnosis, with the added potential of preventing infection. For example, the anti-gag RNA aptamer DP6-12 has been shown experimentally to lower HIV-1 infection (Wandtke et al., 2015). Table 2 summarizes several nanoparticles that can be attached to AS1411 and the conjugates target.

## 5 Conclusion

Nucleolin is a protein overexpressed in many cancers and is used by pathogens to invade host cells and propagate. This allows for the design of targeted therapies and diagnostic tools using the anti-nucleolin aptamer AS1411. AS1411 has been shown to localize and internalize in numerous tumor cells. Once in the cell, it can inhibit nucleolin function and deliver a drug it is conjugated to, allowing for targeted therapy with few off-target effects. AS1411 could also be conjugated to a nanoparticle that allows for the diagnosis of cancer cells at lower concentrations than existing techniques. AS1411 has the potential to do more than deliver drugs or aid in diagnostics. In 2023, the Puzzo lab published their work using AS1411 in gene therapy. They could conjugate AS1411 to an Adeno-associated virus that led to ligand-specific transduction without off-target transduction in peripheral organs (Puzzo et al., 2023). As gene therapy continues to be explored, aptamers such as AS1411 could help to ensure that the therapy limits off-target effects. As research continues, AS1411 has a promising potential to aid in the targeted diagnosis and treatment of a myriad of pathologies.
