# Supplementary material for: Anti-nucleolin aptamer AS1411: an advancing therapeutic
Source: Front Mol Biosci. 2023 Sep 21;10:1217769. doi: 10.3389/fmolb.2023.1217769 (PMC10551449; doi:10.3389/fmolb.2023.1217769)
Supplement: Supplementary file 1 [file DataSheet1.PDF]

Abbreviation	Full-Form
ADP	Adenosine diphosphate
Bax	Bcl-2-associated X protein
Bcl-2	B-cell Leukemia/lymphoma Protein-2
CDC2	cell division control protein 2
CK2	casein kinase 2
DNA	Deoxyribonucleic acid
EBNA1	Epstein-Barr Nuclear Antigen 1
EBV	Epstein-Barr Virus
ECF	Extracellular Fluid
EF-Tu	Elongation Factor-Thermo-unstable
EV71	Enterovirus 71
FDA	Food and Drug Administration
G	Guanosine
G4	Guanosine Quadruplexes
Gag	Group-Specific Antigen of HIV-1
GEF	Guanosine Exchange Factor
HCV	Hepatitis C Virus
HIV-1	Human Immunodeficiency Virus 1
mRNA	Messenger RNA
MUC1	anti-mucin short variant S1
NOD/SCID	Non-obese Diabetic/Severe Combined Immunodeficiency
NS5B	Non-structural protein 5B of HCV
NSCLC	Non-small cell lung cancer
PAK1	p21 Activated Kinase
PAK1	serine/threonine p21-activiating kinase 1
PARP	poly (ADP-ribose) polymerase
PCNA	proliferating cell nuclear antigen
PEL	Primary Effusion Lymphoma
PERS	plasmon enhanced Raman scattering
pH	Potential of Hydrogen
PI3K	Phosphoinositide 3-kinase
pRB	retinoblastoma protein
Rac1	Ras-related C3 Botulinum Toxin Substrate 1
Ras GTPase	Rat sarcoma Virus Guanosine Triphosphatase
RBD	RNA-binding domains
RNA	Ribonucleic acid
rRNA	Ribosomal Ribonucleic acid
RSV	Respiratory Syncytial Virus
siRNA	Small-interfering RNA
Src	Proto-oncogene Tyrosine-protein Kinase Src
Src	SRC Proto-oncogene non-receptor tyrosine kinase
UTR	Untranslated Region
VP1	Virion Promotor 1
WAVE	Wiskott–Aldrich syndrome protein (WASP)-family verprolin homologous protein
WRC	WAVE regulatory complex
